# Challenge clusters facing LCA in environmental decision-making—what we can learn from biofuels

**DOI:** 10.1007/s11367-015-0930-7

**Published:** 2015-08-07

**Authors:** Marcelle C. McManus, Caroline M. Taylor, Alison Mohr, Carly Whittaker, Corinne D. Scown, Aiduan Li Borrion, Neryssa J. Glithero, Yao Yin

**Affiliations:** Department of Mechanical Engineering, University of Bath, Bath, BA2 7AY UK; Energy Biosciences Institute, University of California, Berkeley, CA 94704 USA; Institute for Science and Society, School of Sociology and Social Policy, University of Nottingham, Nottingham, NG7 2RD UK; Rothamsted Research, Harpenden, AL5 2JQ UK; Lawrence Berkeley National Laboratory, Berkeley, CA 94720 USA; Department of Civil, Environmental and Geomatic Engineering, University College London, London, UK; School of Biosciences, University of Nottingham, Nottingham, LE12 5RD UK; EBI, Berkeley, CA USA; Idaho Public Utilities Commission, Boise, ID USA

**Keywords:** Biofuels, Bioenergy, LCA, Policy, Sustainability, Uncertainty

## Abstract

**Purpose:**

Bioenergy is increasingly used to help meet greenhouse gas (GHG) and renewable energy targets. However, bioenergy’s sustainability has been questioned, resulting in increasing use of life cycle assessment (LCA). Bioenergy systems are global and complex, and market forces can result in significant changes, relevant to LCA and policy. The goal of this paper is to illustrate the complexities associated with LCA, with particular focus on bioenergy and associated policy development, so that its use can more effectively inform policymakers.

**Methods:**

The review is based on the results from a series of workshops focused on bioenergy life cycle assessment. Expert submissions were compiled and categorized within the first two workshops. Over 100 issues emerged. Accounting for redundancies and close similarities in the list, this reduced to around 60 challenges, many of which are deeply interrelated. Some of these issues were then explored further at a policy-facing workshop in London, UK. The authors applied a rigorous approach to categorize the challenges identified to be at the intersection of biofuels/bioenergy LCA and policy.

**Results and discussion:**

The credibility of LCA is core to its use in policy. Even LCAs that comply with ISO standards and policy and regulatory instruments leave a great deal of scope for interpretation and flexibility. Within the bioenergy sector, this has led to frustration and at times a lack of obvious direction. This paper identifies the main challenge clusters: overarching issues, application and practice and value and ethical judgments. Many of these are reflective of the transition from application of LCA to assess individual products or systems to the wider approach that is becoming more common. Uncertainty in impact assessment strongly influences planning and compliance due to challenges in assigning accountability, and communicating the inherent complexity and uncertainty within bioenergy is becoming of greater importance.

**Conclusions:**

The emergence of LCA in bioenergy governance is particularly significant because other sectors are likely to transition to similar governance models. LCA is being stretched to accommodate complex and broad policy-relevant questions, seeking to incorporate externalities that have major implications for long-term sustainability. As policy increasingly relies on LCA, the strains placed on the methodology are becoming both clearer and impedimentary. The implications for energy policy, and in particular bioenergy, are large.

## Introduction

Climate change mitigation, energy security and sustainability have become increasingly important in global policy. These have been major drivers behind the increased use of biofuels and biopower in renewable energy portfolios. As climate change and greenhouse gas (GHG) reduction has played a more central role in international policy agendas, policymakers and regulators have looked to existing tools, such as life cycle assessment (LCA) to address new or more complex questions.

LCA has emerged as an approach to quantify and account for environmental impacts in a product life cycle and has served regulatory and permitting needs for decades (Taylor and McManus 2013). When a tool was needed, it was a logical choice, and it now forms a core component of several biofuels policy instruments (i.e. EU Renewable Energy Directive (EU 2009), UK DfT Renewable Transport Fuel Obligation (UK DTI 2012), US EPA Renewable Fuel Standard (RFS2, US EPA 2010) and California Air Resources Board’s Low Carbon Fuel Standard (CARB 2014)). Originally focused on accounting for current or past impacts in existing projects, LCA is becoming forward looking to assess future impacts of a more consequential nature. The methods are slowly evolving as LCA is asked to answer fundamentally different questions.

Traditionally, LCA was used to answer specific questions (Sandén and Karlström 2007) that are directly attributable to the life cycle of a product in the existing technological and economic climate (Sanchez et al. 2012). This is now known as attributional LCA (aLCA). LCA’s newer, undisputedly important task is to help to anticipate future impacts, so as to influence and evaluate policy options as part of governance for sustainability in energy, emerging technology and resources. This is consequential LCA (cLCA) and allows impacts to be considered in a wider, even global, context of producers and consumers (Nuffield 2011). This more consequential approach is considered to be the appropriate method for policymakers (Brander et al. 2009). However, this expansion has been proven challenging and, in some cases, controversial.

LCA is now becoming more heavily relied upon to reveal potential unintended consequences of bioenergy. This use is almost unique to the (bio)energy field and, in some cases, masks a high degree of uncertainty. Since biofuel policies were established in the USA and European Union (EU), there has been concern that unintended consequences could arise and threaten climate and other sustainability goals (e.g. Mol 2007; Jaeger and Egelkraut 2011). Among these have been concerns that a major switch to biofuels produced from food crops will lead to competition between the use of crops for food/feed or for fuel or that using land for bioenergy could potentially contribute to other effects through global markets. There have also been concerns that the production of bioenergy crops being carried out on environmentally sensitive lands, or that land which has been acquired in the Global South, leads to the loss of livelihoods and local food and energy production (van Eijck and Romijn 2008). Further, many of the potential sustainability impact categories for biofuels occur at the systems level and so cannot be easily separated into distinct categories among environmental, economic or social impacts (Mohr and Raman 2013), thus making LCA’s task of anticipating the unanticipated all the more challenging.

Because heavy use of LCA in bioenergy policy entered from the climate discourse, most of the emphasis thus far has been on GHG balances (McManus and Taylor 2015). However, as engagement with broader sustainability standards has grown, the metrics space has begun to broaden to include social and ecosystem impacts among others. While the emergence of such metrics directly serves the policy process, the particular challenge here is that approaches to do so are still in early stages and have correspondingly higher uncertainty. LCA is balanced between its past and continuing future as a data-based, rigorous retrospective tool for product and process optimization and its parallel future as a policy or strategic planning tool.

The goal of this paper is therefore to illustrate the complexities associated with using LCA and the way it is developing, with particular focus on bioenergy, so that its use can effectively inform policy. The analysis draws on expert input from a series of workshops in the UK and USA. It identifies and discusses the specific issues associated with the application of bioenergy/biofuels LCA in a policy context, based on categorizing expert input of challenges arising in the application of LCA. It reflects the three primary stakeholder communities in biofuels/bioenergy LCA: policymakers and other decision-makers who rely upon the results; practitioners and reporters who use the methods for compliance and study; and researchers focused on expansion, development and improvement of the methods. These issues seen in bioenergy LCA are not restricted to bioenergy, but they are being experienced here first due to the global nature of bioenergy production and use and the systemic links between agriculture and energy. Therefore, lessons learned from this area will have implications across both LCA methodology and (energy) policy development.

## Methods

**Fig. 1:**
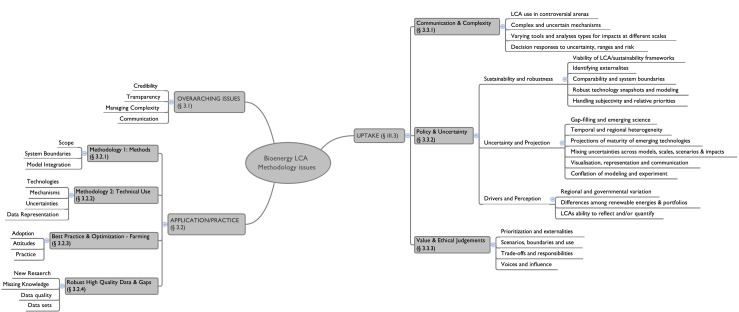
Challenge clusters in LCA for bioenergy

Issues associated with the use of LCA for bioenergy and biofuels were compiled from open expert submissions in association with two workshops run as a result of a partnership between two of the institutions (Bath and Berkeley) in the UK and USA on bioenergy life cycle assessment. The contributors were not restricted in any way in terms of what they submitted, and the group discussed all in depth during the first workshop. Some of the issues raised within these workshops were explored further at a policy-facing workshop in London, UK. The call for issues was deliberately broad to encourage a wide range of contributions but framed as ‘challenges’ associated with LCA in bioenergy. Over 100 issues were contributed. Accounting for redundancies and close similarities in the list, this reduced to around 60 challenges, many of which are deeply interrelated. These were sorted into three supergroups (Fig. 1): overarching issues, application/practice and uptake, each of which is discussed below, illustrating the complexities associated with using LCA and the way it is developing with particular focus on bioenergy policy. Detail and examples associated with the issues identified were provided by the authors through their expert knowledge and literature review.

## Results: issues/challenges

### Overarching issues

There is a generalized set of overarching issues underpinning the policy use of LCA which emerged in the issues submitted: credibility, transparency, complexity and communication. The credibility of LCA is core to its use in policy. Transparency and effective management of complexity and communication are key to this; without them, perception and support falter (Dragojlovic and Einsiedel 2015). Uncertainties and complex, conflicting results reported in studies of the greenhouse gas (GHG) savings from utilizing biomass contributed significantly to major drawbacks experienced in the bioenergy sector over the last decade (Adams et al. 2013).

Transparency across studies is vital for any harmonization of similar analyses; otherwise, large differences can occur, leading to uncertainty (Hennecke et al. 2013). Even small inconsistencies in emission factors used between GHG calculation tools can generate very different results using the same input data. For example, between the ‘BioGrace tool’ and the ‘Roundtable on Sustainable Biofuels (RSB) GHG tool’ for assessing the GHG balance of biofuels, economic operators could enhance the calculated GHG performance of their biofuel by 20–35 % without changing the production process, taking advantage of differences in their emissions factors by selecting the more favourable tool (Hennecke et al. 2013).

A number of regulatory agencies have attempted to overcome this by releasing detailed guidelines for products under their purview (DECC: approval under RTFO; EPA: pathway for RINs under RFS2; CARB: pathway under LCFS). This has resulted in a proliferation of non-transferable guidelines, as each was originally designed for a specific product or purpose. For example, the European Renewable Energy Directive calculation methodology was designed to account for the GHG emissions from biofuels. The allocation method specified in the RED (energy content) cannot easily be employed by those performing similar GHG assessments in other bioenergy systems, for example anaerobic digestion, as the technology generates large quantities of a valuable co-product, digestate, but has negligible energy content (Manninen et al. 2013). As a result, the methods of assessment between each set of guidelines differ considerably in their system boundaries, co-product and waste definitions and methods of allocation of environmental impacts (Whitaker et al. 2010).

These methods all comply with the ISO standards (14040:2006 (CEN 2006a) and ISO 14044:2006 (CEN 2006b)) that govern the LCA practice, but the ISO standards leave a great deal of scope for interpretation and flexibility to the LCA practitioner (Aylott et al. 2011). As a result, it is virtually impossible to make confident comparisons between studies as they can differ not only due to inherent variability between systems but also due to the methods in which they are examined (Menichetti and Otto 2008). This lack of consistency, and hence credibility in places, makes it difficult for government departments and policymakers to use LCA studies (UK Workshop 2012).

There is a great deal of variability and complexity involved in bioenergy system GHG analysis, some of it tied to methodological decisions consistent with standards for LCA and some of it particular to biomass systems. Direct GHG emission means ranged from −56 to 163 g CO_2_eq/MJ of fuel in studies of lignocellulosic ethanol in a recent statistical meta-analysis (Menten et al. 2013), responding to cultivation, yield and soil carbon factors, among others, beyond the obvious differences in feedstock. For GHG emissions from indirect (market mediated) land use change, a summary of midpoint values for corn ethanol (US) from high resolution models ranged from 14 to 82 g CO_2_eq/MJ, reflecting prior land use and management assumptions, as well as global market models (Chum et al. 2011), introducing additional complexity and variability. Even within a coordinated analysis values can differ widely; GHG emissions including indirect land use change comparing uncertainty propagation and different allocation types ranged from about 35 to 85 g CO_2_eq/MJ (midpoints) for wheat bioethanol in the UK (Yan and Boies 2013). In some cases, poor documentation prevents readers from reproducing the results or evaluating the quality of input data; lack of detail and information also results in irreconcilable discrepancies (e.g. Yates and Barlow 2013). In a sampling of more than 40 studies for comparative assessment of rapeseed biodiesel that met a baseline level of detail on method, data and assumptions, only 28 had sufficient transparency for comparison (Malça and Freire 2011).

Failure to transparently report or manage the complexities in bioenergy GHG assessments can lead to confusion and misleading results (Adams et al. 2013) and facilitate misrepresentation that has the potential to focus policies on the wrong areas. The ‘carbon sink or sinner’ report (AEA Technology/Ricardo-AEA 2009) reviewed the sensitivity of the results in the Biomass Environmental Assessment Tool (BEATv2 (AEA Technology and Associates 2010)) to biomass supply chain parameter assumptions, concluding, among other things, that the life cycle GHG emissions were strongly influenced by how the fuel was produced, transported and processed. They stated that ‘bad practices’ involving transporting fuels at very long distances and excessive use of nitrogen fertilizers could reduce the emissions savings for the same fuel by 15 to 50 % (Bates et al. 2009). Collectively assessing these two stages of the supply chain created a misinterpretation that transport emissions were a major cause for concern in net GHG emissions of bioenergy supply chains. Media reports and local anti-biomass websites then reproduced this, emphasizing the less significant transportation impacts rather than nitrogen fertilizer use.

Transparency, relying on detailed communications and chains of evidence, can increase the value of LCA comparisons. However, there are trade-offs for transparency. The level of detail often desired by scientists and practitioners compared with the time available to the policymaker is often conflicting; therefore, in order to support credibility, some mechanism to communicate complexity is required.

### Methodology: application and practice

Issues associated with LCA methodology fell into two categories. The first centers around the formulation of questions for analysis and boundary issues, including models to address these issues. The second set focuses on technologies assessed, mechanisms of impact and data representation.

#### Methodology I: methods: scope, system boundaries and model integration

Goal and scope setting is the first step in performing a LCA and thus influences the subsequent phases (Tillman 2000). It falls to the trained practitioner to enforce the validity of goal and scope setting for the required analyses. For example, the level and quality of data collected must be sufficient in order to fulfill the original aim of the study (Singh et al. 2010). The system boundaries are set during this stage, and these are key to any analysis (Bird et al. 2011). The choice of system boundary in comparative studies may lead to different conclusions and decisions about which products to promote, as in the classic debate surrounding nappies/diapers (Bond 2005), and have an influence on rankings through variation in scope or the impact that is assessed (Suh et al. 2004). Bio-based (poly)lactic acid, for example, out-performed conventional polymers on energy consumption and GHGs; however, clear ranking was lost when ecosystem quality metrics were assessed (Yates and Barlow 2013).

Allocation methods (the way in which impacts are assigned to co-products) give quantitatively different results, which can also lead to incomparability (Wang et al. 2011). An assessment of UK wheat ethanol with regional N_2_O parameters reported GHG emissions (midpoint values) of 51, 64 and 68 g CO_2_eg/MJ for energy allocation based on energy, economics and substitution, respectively (Yan and Boies 2013). This affects both comparison and compliance, as policies mandate different allocation types: by substitution on the RTFO, RFS2, LCFS and RED for electricity co-production, by energy content in RED and as fall back for RFS2 and LCFS and by economic value as a fall back for the RTFO. Thus, the same pathway ranks differently across policies. Direct GHG emissions for bioelectricity from rapeseed using allocation choices from primary renewable policies improved on the reference case by 60 % (sub peas) or 21 % (sub soy) with substitution, 33 % for energy allocation and 16 % for economic allocation (Wardenaar et al. 2012). Such incompatibility can occur even with methodologies compliant with industry or regulatory standards for biofuels GHG reporting (Whittaker et al. 2011).

System boundaries can become inconsistent because of allocation decisions or because the scope or the goals of analysis changes. For example, methods for reducing the overall farm environmental footprint will not necessarily result in a low carbon crop (both of which are assessed with valid LCAs) and vice versa, and the differing purposes lead to different boundaries, which are often conflated (Whittaker et al. 2013). The farm-based approach considers the overall environmental footprint of a farm site, whereas a crop-based analysis focuses on crop- and product-specific aspects that go beyond the farm gate. Agricultural assessments are particularly sensitive to scope changes in response to purpose (Roy et al. 2009), which has major implications for bioenergy policy decisions. For example, the RED was designed to promote the production of biofuels from ‘non-food’ biomass feedstocks and does this by specifying in the GHG accounting methodology that cereal residues are not allocated upstream emissions from cultivation. This immediately places these feedstocks at an advantage but is an example where the intention and approach of the analysis has been heavily influenced by policy (Whittaker 2014). Perhaps more significantly, the detail of goal and scope is seldom conserved when analyses are merged into the policy process, because data, for example GHG values, are taken from a range of studies to inform policy or regulation or to be the basis of financial incentives, such as feed-in tariffs. Often, several studies, which may have been originally produced for a different purpose, are taken to inform policy (McManus and Taylor 2015).

This tendency to aggregate the GHG estimates for comparative decisions is logical but overlooks the complexity and detail specificity associated with GHG analyses for bioenergy systems. For example, Farrell et al. (2006) conducted a meta-analysis of early corn ethanol LCAs and found that, even for direct effects (attributional analysis), the GHG and net energy results varied substantially. Studies with the least favourable results for corn ethanol yielded a carbon-intensity of nearly 120 g CO_2_eq/MJ, which is approximately 30 % higher than gasoline. However, those studies failed to allocate any biorefinery impacts to co-products and used outdated data for key industrial processes. For rapeseed biodiesel in Europe, a careful analysis found calculated emissions ranging from 5 to 170 g CO_2_eq/MJ (Malça and Freire 2011), arising primarily from differences in modelling soil emissions and land use, co-product allocation and high uncertainty associated with some emissions parameters.

Currently, GHG assessments dominate the biofuels sustainability debate; for example, more than half of 53 sampled LCA studies of lignocellulosic biofuels published between 2005 and 2011 were GHG focused (Borrion et al. 2012). Although bioenergy is a global commodity, local values and drivers may differ, and effects like GHG emissions are not always observed near their cause. Location-specific metrics are expanding, though, particularly as water captures more attention and the relevance of other resource competition emerges in the discourse. However, issues other than GHG tend to be specific to the location of production or processes. For example, Scown et al. (2011) demonstrated the location specificity of water consumption/withdrawal and pollution in biofuel production systems. Integrating such impacts is hampered since LCA is, traditionally, not a tool that examines local impacts and thus has crucial gaps. For example, water, particulate emissions and wider impacts on ecosystem services are often not well modelled and may lack spatial or biophysical data. Gaps in data availability and data quality, as addressed elsewhere, are generally not highlighted in the final result, which are often distilled down to a single number (e.g. N_2_O emissions from soil under the IPCC guidelines (De Klein et al. 2006)).

Analyses with complex boundaries often result in multiple models in the final analysis. Ecosystem, market and social dynamics contribute to impact processes. Structural changes, such as infrastructure and technology changes and other progress along the innovation trajectory and adoption rates and patterns, contribute to scenarios for impact and are also beyond LCA’s scope. All of these introduce the need to rely on and integrate increasingly complex and speculative modelling and shape the development of assessment tools (Wicke et al. 2015) and lead to efforts to develop general quality criteria for modelling and recommend best-practice LCIA models for particular impacts from the plethora available (Hauschild et al. 2013). Because advances in elucidating or representing the mechanisms that underpin models occur in many different academic disciplines (see, e.g. Arbault et al. 2014), integrating the sets of data or models to give a picture of the life cycle impacts is challenging, though essential for LCA to contribute policy setting or analysis (Wicke et al. 2015). In connecting models to describe various portions of the overall value chain and potential consequences, the overall complexity and level of detail in the analysis can mask areas where boundaries conflict, even for relatively narrow studies. Ensuring that the full set of models integrate harmoniously and preserve reasonable levels of transparency, rigour and robustness is an ongoing challenge that worsens as use of custom-built tools proliferate (see, for example, CA-GREET 2014).

#### Methodology 2: technical use: mechanisms, technologies and representations of data

System sustainability is often estimated by summing the impacts of value chain components and attributional LCAs (see, e.g. Bento and Klotz 2014). In order to assess the sustainability of biofuel production, it is necessary to consider each biofuel from each feedstock, according to its own merits, and alongside specified sustainability criteria (Royal Society 2008). There is, however, a lack of the necessary codified sustainability criteria and a recognition of data needs to support them (Hecht et al. 2008), and most progression in LCA-based reporting methods has focused on developing a single method (predominantly based around GHG measurement (McManus and Taylor 2015)) to assess all biofuels in a similar manner. The success of this is mixed, especially with the increasing importance of social and economic sustainability parameters, and with mismatches between regulatory mechanisms in methodological approaches such as allocation and default values for fuels and systems.

To be included in an assessment, mechanisms for interactions among portions of the physical system or value chain are also needed. The fundamental interactions are bio/geophysical, such as in soil carbon accumulation and mobility, or in nutrient and water cycles, and give rise to feedbacks at a variety of spatial and time scales (Bagley et al. 2014). These mechanisms draw on scientifically active areas where knowledge is evolving rapidly with both high and low uncertainties (see, e.g. Balvanera et al. 2014; Greene et al. 2015) and provide an idea of the necessary scale of the system boundary needed to capture relevant impacts. Sustainability assessments of biofuel systems show very strong sensitivity to soil emissions, particularly nitrogen. Using different N_2_O emission methods gave GHG values (midpoint) differing by 25 % when using IPCC Tier 1 methods or a UK-specific model within the same assessment of UK wheat ethanol (Yan and Boies 2013). A meta-analysis of LCA studies on European rapeseed biodiesel found that GHG intensities correlated directly with how soil emissions were modelled (Malça and Freire 2011). Some of these mechanisms differ substantially between first generation and advanced bioenergy systems. For example, perennial crops can have belowground carbon allocations more than four times higher than a traditional corn-soy rotation, and belowground biomass increased by 400–750 %, measured for miscanthus, switchgrass and native prairie during establishment ( Anderson-Teixeira et al. 2013). The potential for perennial bioenergy crops to increase soil carbon under some conditions has contributed directly to their preferential ranking in over first generation pathways in policy instruments.

Multi-sector analyses or so-called consequential factors introduce additional complex mechanisms for interactions among the value chain and potential consequences. One route is through mechanisms that do not directly include the primary product. For example, Scown et al. (2014) showed that the net GHG and water impacts of utilizing lignin for heat and power at cellulosic biorefineries or exporting lignin for co-firing with coal varied greatly depending on long-term trends in power plant retirement and new construction. If the export of additional biopower to the grid encourages early retirement of aging coal-fired power plants, the GHG footprint of ethanol in their scenario was predicted to be approximately 50 % lower than if biopower exports encourage deferred construction of new natural gas combined cycle (NGCC) power plants.

Interaction mechanisms can also be through market mediation that emerges within or across borders and/or sectors, which may rely on speculative global market dynamics. Though such consequential approaches started with general product life cycles (Weidema 1993; Zamagni et al. 2012), attempts to assess the impact of global commodity market-driven land use dynamics in relation to biofuels have made it a common concept to guide policy in attempting to avoid unintended consequences in the form of indirect land use change (McManus and Taylor). The drawback is that consensus around the approach has not yet emerged (Marvuglia et al. 2013; Rosegrant and Msangi 2014; Schmidt et al. 2015); thus incorporating such market-mediated impacts for consequential analysis increases the variability dramatically ( Vázquez-Rowe et al. 2013). For example, estimates of ILUC GHG impacts ranged from about 3 to above 220 g CO_2_eq/MJ rapeseed biodiesel and ~5 to ~100 g CO_2_eq/MJ for bioethanol from maize over the last 5 years, even in current analyses when ranges for such values have tightened nor are the authors of that analysis optimistic about such uncertainty decreasing soon (Ahlgren and Di Lucia 2014). Thus far, ILUC has received the bulk of attention among consequential LCA of biofuels. But market-mediated mechanisms are also being explored for other properties, such as indirect fuel use (Rajagopal et al. 2011) and rebound effects (Vivanco and van der Voet 2014; Smeets et al. 2014). Likewise, social impacts, crucial for sustainability assessments, arise through cross-cutting mechanisms (see, e.g. Benoît et al. 2010). About 20 % of annual LCA publications touch on or address social factors (McManus and Taylor 2015) and guidelines and methodologies are developing broadly because of the labyrinthine relationships among groups and the range of potential impacts (Wu et al. 2014). Many of these are reflected qualitatively in international sustainability standards (see, for example, FAO’s Compilation of Bioenergy Sustainability Initiatives 2011). In nearly all these mechanisms, the science or state of knowledge is changing rapidly. As new insights emerge, accounting for impacts in the LCA that depend on them is lagging.

The absence of spatiotemporal components, which underlie most mechanisms in LCA, is of particular concern for bioenergy. Time is most commonly incorporated with set-year analyses (e.g. N years in the future) and linear annualization. While the former is a viable, if limited, approach, the latter is problematic for agriculture. Simple annual crop rotations that make up the bulk of agriculture (corn, soy, wheat, etc.) are well represented this way, but, for perennial crops, where yields develop over time and management practices may vary from year to year, simple annualization is not always reflective of reality. The difference between establishment and production periods for such crops are key to emissions estimates; simulations of nitrogen loss from 2-year-old switchgrass were 360–410 % higher than for mature stands at the same fertilization levels, depending on harvest number, resulting in a 15-year average of 20–30 % that of cotton under the same conditions (Sarkar et al. 2011). Likewise, the temporal mismatch between conversion or harvest and carbon uptake in forest-based resources introduces decadal and longer time frames, and concerns over the potential carbon debt caused between harvesting and re-establishing timber stands have become an important issue for climate and bioenergy policy (Lamers and Junginger 2013).

Location-dependent issues for LCA are not limited to siting information and land use changes; they are dominated by the limitations in inventories from location dependence in input data and the location dependence of impact metrics water or biodiversity (Seager et al. 2009) as well as trade, market mediated and social impacts, all addressed in other sections. Spatial characteristics of the feedstock production system are often assessed extraneously in economic terms. Scown et al. (2013) found that, in their scenario that incorporated corn stover, wheat straw and Miscanthus in the USA, only 80 % of available biomass was geographically concentrated enough to warrant utilization for biofuel production; the remaining biomass was too dispersed, resulting in prohibitively high transportation costs. While highly sensitive to supply chain characteristics, LCA is still evolving to incorporate the intersection between developing infrastructure and logistics, such as transportation modelling (Strogen and Horvath 2013; Strogen and Zilberman 2014). The spatial aspects of resource management will also begin to contribute in bioenergy LCA, as in critical resource assessment (Sonnemann et al. 2015), potentially to pivotal effect, because there are more critical resources than just land in biofuels, and land is a critical resource in more than biofuels. Generally, these issues introduce reliance on multi-model, multi-scale systems. Data integration, uncertainty integration, error propagation, certainty and multi-parameter output representation all become important and are difficult to convey succinctly.

#### Best practice and optimization—farming

Agriculture and farming in particular deserve special mention as competition for land increases. Financial incentives awarded to the farmer to encourage planting of bioenergy crops create an interaction among LCA, policy and farming practices (Glendining et al. 2009; Natural England 2013), as does the global commodities feed/food market. The effectiveness of such incentives to serve climate policy goals depends on the accuracy of calculated avoided life cycle GHG emissions and the system boundaries considered. Farmers are key to the provision of empirical data; increasing the certainty of the assessments for that portion of the life cycle. However, because agricultural practices differ by region, even over relatively small distances, and management decisions have large impacts in bioenergy (Davis et al. 2013), transferability of data or existing analysis from a region where much is known to one where little is known (a common technique in other sectors) is questionable.

LCA for agricultural production is well-established (Roy et al. 2009), but LCA for policy planning in bioenergy incorporates the potential implementation of large-scale biomass production, which lacks certainty. Improvements in agricultural yields have been substantial over the past decades (e.g. US corn yields have roughly doubled since 1976, USDA data). Projections of productivity changes over time are speculative but important for long-term planning. For example, carbon payback times decrease 30–50 % when crop yields reach the 90th percentile in global yield, representing crop productivity increases and/or substantial management changes across global averages (Gibbs et al. 2008), in effect identifying the benefits of addressing the global yield gap. There are potential benefits in non-GHG impacts also. For example, in advanced bioenergy landscapes, it is possible for very small trade-offs in the economic balance to have a large favourable impact on biodiversity under particular land conservation regimes (Evans et al. 2015). These represent challenging aspects for cross-sector agricultural LCAs to include. Other complicating factors that are of increasing importance include conservation mechanisms which impact biodiversity (see, e.g. Evans et al. 2015), precision agriculture where practices vary between or even with fields (Weekley et al. 2012), alternate cropping systems (Perrin et al. 2014) and land sparing practices (e.g. Cohn et al. 2014) which are often multifunctional. Multifunctional landscapes provide many sustainability benefits (Perfecto and Vandermeer 2010) but expand the system boundaries increasing data requirements (Rossing et al. 2007) and uncertainty (Jung et al. 2013).

Risk, policy uncertainty, nascent markets, infrastructure and supply chains threaten adoption, which makes projection of roll-out rate or the extent of production challenging. This increases reliance on representing statistical projections in data and uncertainty and shifting policy estimates of expansion (Rajagopal and Plevin 2013), including how to reflect the ranges in the results, and whether knowledge of the demographics can contribute to analyses or types of analyses that are more useful from a policy perspective (Plevin 2010).

#### Robust high quality data and gaps

Data availability, transparency, curation and sharing are key to the long-term success of LCA both generally and for comparisons used in bioenergy planning. Reliability of the results from LCA studies strongly depends on the extent to which data quality requirements are met, where common problems include lack of transparency and data variation and gaps. For lignocellulosic ethanol, for example, data inconsistencies contribute to conflicts in published LCA results of GHG emissions (e.g. Wiloso et al. 2012), ranging from −1.25 to 0.84 kg CO_2_eq/km travelled when using E100 (Borrion et al. 2012). There are specific cases where there is minimal data, for example, enzyme production (Spatari et al. 2010), or where data is outdated, for example, pesticide production (Audsley et al. 2009). Primary data sets, despite their overall strength, also sometimes have errors (see, e.g. Cooper et al. 2012). Data set compositions vary regionally, and sometimes sectorally, in the completeness of their data on which GHGs are included (Ansems and Ligthart 2002). Reusability of data is highly dependent on sufficient data documentation, and standards are emerging (see, e.g. Sonnemann et al. 2013) to address this, for example, in the area of LCAs used for Environmental Product Declaration (EPDs) (Ingwersen et al. 2012).

While transferability is a common approach to supply missing data, using the value from another region for a missing parameter, the approach frequently fails for bioenergy. Data for non-global environmental impact categories frequently vary by region, often for categories to which agricultural assessments are highly sensitive (e.g. ecotoxicity). Some impact categories, such as water quality and use impacts in bioenergy, can be local, regional or both (Dale et al. 2010) and are further complicated by the importance of the local water context (e.g. drought to excess). Water consumption, for example, may range from 5 to 2138 L per litre of ethanol depending on regional irrigation practices in the USA (Chiu et al. 2009)). Region-specific data are also often not available (for example land use and biodiversity (Dale et al. 2010)). Industrial data for emerging processes and technologies is frequently limited due to commercial confidentiality and also as the data for such systems and an understanding of their impact is nascent. New scientific understanding of various impacts, such as the impact of air pollutants on human health (see, e.g. Hajat et al. 2015; Novák et al. 2014), or newer issues such as ‘black carbon’ (see, e.g. Cai and Wang 2014; Otto et al. 2014), also introduce data or method limitations.

Under some conditions, data sets/databases used in a LCA reflect external value judgments, of which users may or may not be aware or take time to discover. For example, there are levels of detail within and between datasets, some with wider boundaries than others. There are also value judgments embedded in ‘off the shelf’ impact assessment methods. Some are more explicit than others, for example EcoIndicator 99 and ReCiPe both show three options ranging from an ‘absolutely only proven cause and effect’ to a precautionary approach. Pragmatically, these databases and software packages are frequently the most accessible solution for the practitioner since primary data is either unavailable or too time consuming to gather (Hetherington et al. 2014). This is reflected in the literature, where uptake of these databases is rapid; studies citing the widely used ECOINVENT database launched in 2000 (Frischknecht et al. 2004) had reached 2240 by mid-2014 (scopus search, 18th July 2014). However, these proprietary databases and software tools can limit transparency, independent reproducibility and transferability as the underlying data cannot be shared in publications. The high-cost setting up and maintaining the quality of database impedes open access (Hellweg and Canals 2014). A number of open data sets and standardization efforts seek to address this, in particular the European Platform on LCA’s European reference Life Cycle Database and the International Reference Life Cycle Data system common data format standard, as well as and the USDA LCA Commons and NREL US Life Cycle Inventory Database.^1^

Because a wide range of stakeholders use the results, indications of data quality are increasingly important. Qualitative indicators can be used along with their quantitative counterparts to address data transparency issues such as data source and to address the confidence and uncertainty of data (see for example, Ansems and Ligthart 2002) under particular circumstances, such as in the electricity sector (see, e.g. Garraín et al. 2015). Qualitative indicators help to contextualize the relevance of the data so that policymakers are able to make more informed decisions about the circumstances to which the data apply, thus making policy judgements based on the available data more reliable. Such indicators are sometimes contained within commercial software packages (for example SimaPro—Pre Consultants (2014) and GaBi - PE International (2014)). While these packages can aid very quick and effective calculations, it is also very easy for general users to employ them and default databases without an understanding of the uncertainty or quality of the data contained within them.

### Uptake

Uptake of LCA results has been primarily driven by policy setting in recent years, a change from its prior regulatory use (McManus and Taylor 2015). Three main areas are associated with uptake: communication and complexity, policy and uncertainty and value and ethical judgments (Fig. 1). These are explored in detail in the following sections.

#### Communicating scientific complexity and uncertainty

Communicating scientific uncertainty is relevant both within the LCA community itself, where practitioners often disagree in terms of practice and culture, and with an increasingly broad range of stakeholders, such as the public and policymakers. Given the growing use of LCAs to help guide decisions in controversial topics cutting across different policy domains, such as bioenergy, communicating the inherent complexity and uncertainty becomes more challenging and important. A key component for policy in these controversial topics is that often there is no single solution. For example, geographical and/or temporal dimensions of sustainability impacts produce uneven effects, magnified by the globalization of biofuel production and trade. Awareness of these global impacts is growing; at the same time, local impacts are becoming more widely known (Raman and Mohr 2014). Other tools also address these, such as Environmental Impact Assessment (EIA), and a variety of footprinting with a range of indeterminacies (Andrew et al. 2009; Johnson and Tschudi 2012; Newell and Voss 2011). Policymakers may have varied knowledge of what is, or is not, encompassed in the assessment approach.

At the highest level of detail, the evolution of issues included in an LCA is effective, but at the broader level, the evolution is less smooth. These limitations need to be communicated. Ranges in results and standard uncertainty in data decrease confidence in the results beyond the LCA community. Policymakers generally rely upon straightforward, pragmatic information and while they also require transparency, they often do not feel comfortable with communication at the level of complexity that LCA practitioners feel is necessary. Uncertainty could be considered to be a weakness in LCA, particularly where a ‘single answer’ may be considered by some to be preferential. Through use of the correct tools, however, uncertainty assessment can in fact be used to help support the results by providing a more comprehensive account of the likely range of results (Björklund 2002) and a comprehensive understanding of the problem and its possible solutions (Hellweg and Canals 2014). For example, triangulation with qualitative data enables policymakers to consider quantitative assessments in the broader context of social judgements on what is considered important and why.

Controversial topics often also invoke different value sets that vary by community and region (Walls et al. 2005; Corner et al. 2014), as seen in bioenergy projects. Analysis of the past management of such issues and differences in the outcomes/policy measures may provide insights to assist in bioenergy governance and its use of LCA. For example, genetically modified organisms generally see policy support in the USA and opposition in the UK/EU (Mohr et al. 2012; Marques et al. 2014), while climate agendas show the opposite trend (Howell 2013; Corner and Randall 2011). Where successful, it is likely that broader value-based questions were taken into account (Howlett 2012), and engagement before technical and policy positions became entrenched contributed to success (Mohr et al. 2013).

**Fig. 2:**
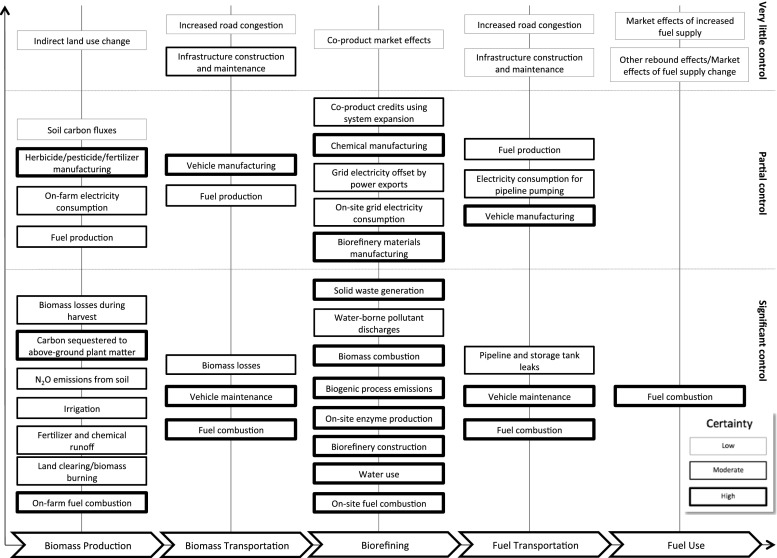
Areas of uncertainty in the bioenergy life cycle

The presence of uncertainty creates challenges in assigning responsibility and thus accountability. For the biofuels/bioenergy supply chain, the impacts that occur at each stage vary in certainty and in the level of operator control, as illustrated in Fig. 2. For each stage, there are varying levels of operator control, ranging from issues such as indirect land use (very little operator control) to water use in a bio-refinery (significant operator control). For example, on farm, fuel combustion is both controllable (through machine production, selection and driver control) and the emissions associated with it are relatively certain. Areas of low certainty are often those where there is little control, such as indirect land use change (ILUC). Many of these are considered both ethically important and challenging in the policy process (Nuffield 2011).

As shown in Fig. 2, these levels of certainty and control also have implications at each stage in the LCA process. Because the value chain consists of distinct but (inter) connected sections, LCA’s inclusion of these can vary. This may be most apparent in the preliminary description, where some components may contain sections that are well described with existing data, to aspects of the value chain that are based on more speculative technology where data may be either highly variable or estimated. In order to move from inventory to impact parameter, both a mechanism and responsibility must be represented. Variability in how well either is known may or may not be reflected in the output. From a method-development perspective, research practitioners are aware of these issues but that this area of the LCA method is under constant development which may not be apparent outside of the practitioner community. Similar issues arise where LCA integrates with other tools, such as in the representation of market-mediated effects. In such cases, there may be variation in mechanism or model for inclusion and variation in data drawn on, as well as differing connection to other tools. The framing that underlies the analysis also reflects decisions about what is controllable, who has responsibility and how necessary it is to include components that have higher than average uncertainty associated with them.

Because policy setting is often concerned with comparing across several options, lack of comparability at best leads to fortuitous agreement and at worst erroneous conclusions and decreased confidence in the analytical community. Use of results in different communities does not always bring the originating community’s caveats with them, increasing the challenge of using LCA studies across disciplines. For bioenergy, these caveats can include describing the range of results may have, either from modelling or from lack of/variability of data, assumptions about time horizons and projection differences across studies, and situations where the original uncertainty range is large. Subjective factors, or those where causality is not fully established, also contribute to policy development, but the challenges around these factors are seldom communicated effectively. As a result they are either ignored or taken as concrete, both of which make ranking and comparisons problematic.

#### Policy and uncertainty

Bioenergy policy-making relies on projecting forward new technology, development and use because it is concerned with future courses. Such projections draw on assumptions about future economic growth, fossil fuel prices, energy generation costs, population and similar variables that are often implicit or embedded in a separate analysis (see, e.g. use of IEA scenarios in projected GHG emissions from energy) and on technology performance projections. These projections of maturity of emerging technologies are inherently uncertain and involve, among other things, assessing potential learning, changing impacts with scaleup, structural change and adoption (Hetherington et al. 2014). LCA can incorporate learning developments by estimating differing efficiencies along the supply chain, and, with some caveats, scaleup by drawing upon estimates for efficiencies of scale, process modelling and industry experience (Lloyd and Ries 2007; Hetherington et al. 2014). However, the difference between measured data and modelled numerical estimates is easily blurred and the two are often treated as interchangeable (Youngs and Somerville 2014). Prospective analysis, where future technologies are assessed, will have higher uncertainty than retrospective analysis than models existing or past production systems (Kendall and Yuan 2013). For biofuel policy, these prospective analyses are common; where they include indirect, or market-mediated effects, the uncertainty increases significantly (Kendall and Yuan 2013).

Drivers for and perceptions of both biofuels and bioenergy vary globally. Some countries, such as Brazil and Scandinavia, have high levels of bioenergy due to funding and acceptability, among other factors (see discussion in for example, Ericsson et al. 2004; Walter et al. 2012, and Lynd et al. 2011). The motivation and drivers in these areas are different from those in regions where bioenergy has lower public acceptance (see, e.g. Dragojlovic and Einsiedel 2015). Consequently, how policymakers frame questions to be analyzed with LCA and prioritize impacts or outcomes differ, reflecting these drivers, perceptions and local constraints. This is relevant particularly for biofuels moving in international trade or subject to international sustainability standards.

The viability of current sustainability frameworks to support policies internationally still needs to be determined. Likewise, the extent to which LCA contributes to quantifying or differentiating among issues related to perceived drivers and barriers in countries with substantial biofuels or bioenergy trade (especially corn and sugar ethanol, pellets and biodiesel) needs to be codified to support compliance with international agreements. Currently, LCA is used as a static tool, while being asked dynamic questions. Forward assessment of sustainability impacts using LCA lies at a complex interface among applied and basic science, engineering and crucially policy. It is also both coupled and dynamic; all of these aspects act on each other simultaneously.

#### Value and ethical judgments

Value judgments are often taken as distinct from the overall LCA process; however, they may have a material effect on the results of the analysis. They can shape what is included within the boundaries of an analysis due to a combination of concern over and knowledge about a particular issue. Through selection of specific boundaries, impact categories or assessment methods, they can also focus analyses to a single location or problem, when in reality, observing regional and sectoral variation may provide a more complete or practical assessment, which may have a strong influence on the final results if crucial impacts are not included (Whittaker et al. 2013). Determining ISO-appropriate boundary criteria (CEN 2006b), however, requires some prior knowledge of the system. LCA production and evolution is influenced by different voices, with a range of familiarity with LCA as a technique or with what is needed by those who rely on the results. This has begun to change the shape of the tool, its use and interpretation. Once the purview of trained practitioners answering specific questions, LCA is now increasingly used to address more broad questions posed by users with less expertise (Whittaker et al. 2013). There is apparent confusion over the role that LCA for one specific question has over addressing broader concerns (Brander et al. 2009). Value judgments, thus, can be a serious issue when LCA is used for comparative analysis of scenarios that will guide policy decisions. For example, the European Commission aims to address indirect land use change in calculations involving biofuel regulation; however, it could be argued that this is not solely a bioenergy issue (Adams et al. 2013).

The way in which value judgments are prioritized raises ethical questions, particularly as they are frequently implicit. For example, which impacts to include in a study, the choice of functional unit and system boundaries may be driven by balancing trade-offs where groups, areas or communities are affected differentially (Bustamante et al. 2014; Calvin et al. 2013). Capturing these trade-offs is what creates challenges and opens new space for LCA methods as they develop. Decision ranking reflects the framing values (Taylor 2012). How effectively they can be included depends on uncertainty, which is fluid term in this particular context. It thus affects the support for including ethically important but less concrete factors in the analysis.

## Discussion and recommendations

In its current, and currently changing, form, LCA is striking a precarious balance among being a stable product improvement tool used by companies, and/or a policy or strategic planning tool, and a scientifically based rigorous and ever-improving method. It is, in essence, serving somewhat conflicting purposes: product improvement or measurement and policy development (retrospective and improvement, or future orientated). While the challenges identified in the expert poll cluster into a few main areas: communication and complexity, policy and uncertainty, value and ethical judgments and application and practice; many issues are found in multiple clusters and are interrelated. Those issues found in the most clusters and with the most connections in and beyond their primary cluster are high-priority targets for research, development or outreach. The high density of issues in application and practice categories suggests that the approach is still technically immature, and filling those gaps will help significantly.

LCA’s credibility is essential for its use in policy. It is becoming increasingly apparent that even LCAs that comply with ISO standards and policy and regulatory instruments leave a great deal of scope for interpretation and flexibility* (see discussion in Ahlgren et al. 2015). The aggregation of discrete, independent studies into comparisons to inform policy introduces often-invisible inconsistencies from disparities in system boundaries and the judgments made within the original studies. The resultant lack of consistency undermines the method’s credibility, making it increasingly difficult for policymakers and government departments to rely on the results generated by LCA. Nevertheless, LCA is becoming more commonly used within regulation and (energy) policy. The changes in demands on LCA for bioenergy assessment will appear in other sectors and are likely to emerge into other policy areas incrementally, as is already beginning to happen for bio-based products in Europe (Fritsche and Iriarte 2014).

As LCA use expands in policy and regulation, custom-built tools proliferate. These are particularly common to agriculture and bioenergy (e.g. carbon calculators (Kim and Neff 2009; Newell and Vos 2011)). While there are advantages to these, they are strongly sensitive to the data embedded within them; even minor discrepancies can cause considerable differences in the determination of GHG impacts of energy systems. Larger differences based on the framing or scope of the tools, especially among tools based in different regions, have even more significant impact for planning and policy. Acknowledging this and ensuring consistency in underlying data and models is key for effective policy use.

Policy for and management of bioenergy in the broader context of sustainability has emerged more virulently than for energy or agricultural policy generally. While these issues are starting to emerge in other sectors, they have particular urgency here. Stakes in the energy debate are very high, because change in the energy system is connected to climate impact and to economic growth. It has created a driver for policymakers to ask for quantification of these impacts. It has also driven the use of LCA into a new area: a more consequential, policy-making and governance approach to LCA.

Projections of future economic growth, fossil fuel prices, energy generation costs, population and future climates are all (often implicitly) made as part of the policy-making process. All of these projections are uncertain. LCA is used as part of this process to measure the impact of the technologies or the consequences of the policies. It is an exciting approach, but those using the results and tools often are not fully aware of the uncertainties associated with what is often seen to be a tool that can give a definitive result. Its long-term viability as a tool to inform policy still needs to be determined. It is a predominantly static tool, being asked to answer ever more dynamic questions.

Based on the expert-submitted issues, the process of categorizing them, and their relationships, a number of things would make this process both smoother and more effective.

Recommendations:

- Take steps to modulate the discourse by investing in and supporting efforts to integrate the communities (attributional, consequential, scientific and end users) and support outreach to promote awareness of methods’ maturity levels, capabilities and developmental efforts, inter alia;
- Carry out high-level, coordinated model intercomparison activities with common scenarios for commonly used tools, as has been done in climate modelling;
- Develop and support open global, centralized, curated databases, even if semi-sector specific, with public application programming interfaces, and support for and outreach about national level databases;
- Promote caution in the proliferation and use of custom-built tools (such as carbon calculators) and encourage best-practice development in their production through the use of open, centralized datasets;
- Take steps to increase proficiency among general users of the few market-leading software packages, to reduce the errors and lack of reproducibility, transparency and transferability through outreach campaigns to increase awareness and supporting low-cost training initiatives;
- Modify/develop new reporting standards for consistency in consultation with international decision makers, to indicate projections, uncertainty, boundaries, comparability and barriers to comparability;
  - Strive for a ‘quick-view’ graphical representation to be aligned with that from another study so that it is immediately apparently where they are not consistent;
- Commit to and invest in active method development to better incorporate spatiotemporal factors (e.g. projections) and integrate with other impact or sustainability models; and
- Commit to and invest in substantial scientific and social sciences research to address data gaps, supported by engagement activities from the LCA communities and decision makers to identify crucial, high-impact on decision areas.

The recommendations above are clearly appropriate for policy use of LCA beyond bioenergy as well. Our compilation and assessment of issues highlights several learnings for policy use of LCA that transcend bioenergy and may help adoption and use in other areas to be less contentious and more effective. The highest-impact transferable learnings are around: open communication, loss of sector separability, managing data availability and quality, more holistic metrics, evidence-based governance and accountability and context-aware framing.

The bioenergy case has demonstrated that disconnects in communication at each level across disciplines (science, engineering, economics, impact assessment, policy) and with stakeholder groups limits uptake of new understanding and restricts measured evaluation. Uncertainties and the failure to convey the limitations of available data or assessment capabilities, both in bioenergy and in systems it is compared with, contributed to a highly polarized response to bioenergy and obscured the decision space. The integration of the energy and agricultural sectors in bioenergy LCA is demonstrating the need for bigger system boundaries to avoid leakage and suggesting that there may be integrators preferable to LCA. The frequent failure of transferability to fill data gaps in bioenergy is indicating where similar problems will arise beyond bioenergy, particularly for calculating time- or space-dependent impacts or for location-specific aspects of the value chain.

GHG indicators have been a wedge toward more holistic metrics and quasi-metrics in bioenergy LCA, driven by sustainability standards and regulations, a trend to be expected in other sectors. Bioenergy LCA and regulation are exposing snags in using LCA to provide support for evidence-based governance in the absence of causality or operator control. Where land use impacts occur beyond borders and are market-mediated rather than direct, consensus around modelling and inclusion has been slow to emerge and been contentious. Bioenergy LCA is demonstrating that large-scale global aggregate impact assessments (in any area) need coordinated activities or should be approached with caution. Because drivers for and perceptions of bioenergy/biofuels vary globally, local policymakers frame questions to be analyzed with LCA differently, producing differing scope/boundaries and thus differing rank ordering of outcomes, which in turn makes it difficult to assess aggregated global scenarios.

## Conclusions

Bioenergy policy-making is fundamentally a future-oriented, globally aware activity. The emergence of LCA in bioenergy governance lends itself to being something we can learn from because other sectors are likely to transition to similar governance models. As the complexity and breadth of policy-relevant questions have expanded, LCA is being stretched to accommodate them, seeking to incorporate externalities that have major implications for long-term sustainability. And as policy increasingly relies on LCA, the strains placed on the methodology are becoming both clearer and impedimentary. The implications on energy policy, in particular bionenergy, are large. The research clearly indicates not only that we need to ensure robustness and transparency but also that the tool we have stretched further and further requires more before it can be used to model such complex markets.

Many of the issues discussed here are not new but are gaining increasing importance as LCA becomes a more widely used tool within regulation and policy. This has led to some discussion as to whether LCA is fit for purpose and whether the results from an LCA can reliably support policy and planning. It is interesting to note that in the very first issue of the International Journal of Life Cycle Assessment, authors were already asking “Can LCAs fulfill the high expectations placed on them as aids to decision-making in the policy sector?” (Schleicher 1996). This question is asked by practitioners and policymakers more and more, and the answer will become more important as LCA becomes more embedded in policy decision support.

## Footnotes

1 EPLCA: http://eplca.jrc.ec.europa.eu/; ELCD: http://eplca.jrc.ec.europa.eu/ELCD3/; ILCD Standard: http://eplca.jrc.ec.europa.eu/?page_id=86; NREL USLCID: http://www.nrel.gov/lci/; LCA Commons: http://www.lcacommons.gov/
